# A real-world dataset of group emotion experiences based on physiological data

**DOI:** 10.1038/s41597-023-02905-6

**Published:** 2024-01-23

**Authors:** Patrícia Bota, Joana Brito, Ana Fred, Pablo Cesar, Hugo Silva

**Affiliations:** 1 Instituto de Telecomunicações, Avenida Rovisco Pais 1, Inst. Sup. Técnico, Torre Norte, Piso 10, 1049-001 Lisbon, Portugal; 2 Instituto Superior Técnico, Dep. of Bioengineering, Avenida Rovisco Pais 1, Inst. Sup. Técnico, Torre Norte, Piso 10, 1049-001 Lisbon, Portugal; 3 Centrum Wiskunde & Informatica Amsterdam, The Netherlands & Multimedia Computing Group, 2600AA Delft, The Netherlands; 4 Delft University of Technology, 2600AA Delft, The Netherlands

**Keywords:** Biomedical engineering, Human behaviour, Scientific data

## Abstract

Affective computing has experienced substantial advancements in recognizing emotions through image and facial expression analysis. However, the incorporation of physiological data remains constrained. Emotion recognition with physiological data shows promising results in controlled experiments but lacks generalization to real-world settings. To address this, we present G-REx, a dataset for real-world affective computing. We collected physiological data (photoplethysmography and electrodermal activity) using a wrist-worn device during long-duration movie sessions. Emotion annotations were retrospectively performed on segments with elevated physiological responses. The dataset includes over 31 movie sessions, totaling 380 h+ of data from 190+ subjects. The data were collected in a group setting, which can give further context to emotion recognition systems. Our setup aims to be easily replicable in any real-life scenario, facilitating the collection of large datasets for novel affective computing systems.

## Background & Summary

In recent years, the field of affective computing i.e. “computing that relates to, arises from, or influences emotions”^1^, has gained prominence with more than 10k papers published between 2021–2023 (Scopus: searching for emotion and affective (https://www.scopus.com/ in the last 3 years). The literature has observed a growth in text sentiment analysis and image/video-based emotion recognition through body posture and facial expression. For this type of research, large data corpus are available (e.g. AffectNet^2^ with 0.4 million annotated facial expressions; EmotiW challenge^3^ with 1088 annotated videos), which is crucial for the development of accurate artificial intelligent algorithms. In addition to body and facial expressions, emotions can be measured using autonomic nervous system responses.

This growth has also been observed in emotion recognition based on physiological data, namely through unobtrusive physiological sensors which aim to capture data in real-life settings. Compared to video or text-based emotion recognition, the use of physiological data offers several advantages, including unobtrusive data collection over extended periods for individuals and groups, high spatial and temporal resolution, and reduced susceptibility to conscious manipulation by subjects.

In the physiological-based affective computing literature, there is a large number of public datasets available (e.g.^4–10^). However, the majority of these datasets are designed for data collected in the lab and rely on the use of short clips/images validated to elicit basic emotions, e.g.^5–7,11^. The use of short video clips does not replicate a naturalistic emotion elicitation setup where normally we can see a build-up of emotion. This method can introduce bias, as the anticipated emotion may be mistaken for the ground truth instead of the self-reported one. Such a scenario is plausible especially when the intended emotional response to a clip is noticeable, potentially causing individuals to confuse the expected emotion with the one experienced. Moreover, utilizing self-reported emotions to categorize entire clips, ranging from a few seconds to minutes, obscures the dynamic nature of emotional responses throughout the clip’s duration.

While in-lab experiments allow higher control over the collected data, the literature has questioned whether they can be replicated and generalized to real-life scenarios^8,12^. The authors in^13^, compared stress responses induced in the lab to stress induced at the volunteer’s home. The experimental results showed that the volunteer in-lab HR during the stressor was lower than in the real-world setting. Similarly, in^14^ the authors observed that physiological data collected in the lab differed from data collected replicating real-world data, where the person is free to move. This resulted in the test of a model created in the lab showing a deterioration in emotion recognition accuracy when tested with real-world-like data.

A further example of the limitations of in-lab data collection setups is the US government^15^ MOSAIC program, which had the goal of evaluating affect detection systems in real-world scenarios. The data was collected following a lifelog setup, during everyday routines, such as working and work-related travelling. Emotion-related ground truth was collected for positive and negative affect, stress and anxiety. These were measured at the start of the study and once per day using the ESM technique. Neither of the teams met the program goal metrics for affect detection, attaining accuracy near zero across teams. Based on the results, the authors in^15^ suggest the need for a different data collection paradigm that is closer to a real-world scenario.

The terms “in-the-wild”/“real-world” or “naturalistic” data have been denoted to describe data collection when the experimenters do not control the emotion elicitation nor constraint the data acquisition^12^. These can be further divided into “ambulatory” settings where the data is collected in daily living with the subjects moving freely, or “static” when the data collection is limited to a specific location (such as workplace, car or cinema)^12^.

The state-of-the-art has been moving from in-lab controlled and small-video excerpts setups to alternative data collection approaches closer to naturalistic scenarios, such as the BIRAFFE2^16^, in which physiological data was collected (ECG, EDA) during video games, or the PPB-Emo dataset^9^ with physiological data collected (EEG) during driving.

A limited number of robust datasets with data collected in real-life scenarios can be found, such as the DAPPER^8^ and K-EmoCon^4^ datasets. The DAPPER dataset^8^ follows a lifelog paradigm, with physiological data (HR, EDA, and accelerometer) collected during the volunteers daily living for five days. The data was annotated using the ESM and DRM techniques. The ESM was performed 6 times a day, asking for the momentary emotional annotation on the volunteers’ smartphone; and DRM was performed at the end of the day asking the volunteers to recall and annotate the major emotional and behavioral episodes throughout the day. The K-EmoCon dataset^4^ contains physiological data (PPG, EDA, HR, EEG) collected across naturalistic conversations, namely paired debates on social issues. The data was annotated retrospectively by the participants, their debate partners and themselves by watching their recorded facial expressions and upper body data.

In this study, we fill this gap by providing a labeled naturalistic dataset designed for group emotion recognition — the G-REx dataset. In line with the naturalistic data collection, we rely on real movies for emotion elicitation, using physiological sensors integrated into an unobtrusive and wireless bracelet device, and collect data in a group setting (analogous to a cinema theatre). This paradigm allows for a naturalistic emotion elicitation over a long period (each movie has around 2 hours). In this paradigm, we collected data from over 190+ subjects, covering 31 movie sessions and more than 380 hours of physiological data. Our proposed experimental setup for annotated affective data collection can be replicated across diverse naturalistic experimental settings, from a cinema session to a classroom or a hospital.

The collected data can be used for the implementation of affective computing systems across diverse applications, such as neurosecurity systems to warn the users of emotion-manipulative content, modulating live-performances/media content to the audience response or development of subject-specific recommendations^17^, among others.

### Emotion measures

Taking the componential view of emotion^18^, emotions are multi-component emotional responses to relevant stimuli for the subject survival or well-being. This multi-component emotional response comprises changes in the individual subjective experience (feeling), peripheral signals, central physiology and behavior. Central physiology relies on the use of specialized machines such as functional magnetic resonance imaging (fMRI), or positron emission tomography (PET) for emotion assessment. These imaging techniques allow a high-resolution view of the brain region activated by different emotions. However, they have strong drawbacks to be used daily, such as requiring specialized facilities, being large in volume, having an elevated economic cost, requiring the presence of a trained technician, and requiring the individual to remain motionless.

An alternative is the analysis of bodily behavior for emotion recognition. The analysis of facial expressions is one of the most studied areas in affective computing as computer vision techniques can be used for affective computing applications and the collection of ground truth is facilitated. However, they depend on the subject externalization of their emotional expression and are to be recorded by a camera at all times for daily data collection. A third approach, commonly used in clinics is the use of self-reporting. Self-reporting can be expensive and the subject might not want to disclose their true emotions. Nonetheless, self-reporting is often the ground-truth method for the development of automatic emotion recognition algorithms.

Physiological signals such as EDA and PPG can bypass these constraints since they are acquired by very small sensors through a single contact with the skin in unobtrusive places such as the wrist or hand. These characteristics facilitate the collection of large amounts of emotion-related data in the real world.

Other physiological signals such as EEG or ECG also contain information for emotion recognition. However, their use is not as favorable for daily use as the former requires the use of gel electrodes on the hair, and the latter a two-point contact.

Peripheral signals are controlled by the ANS, namely the SNS and the PNS. The SNS is often referred to as the “fight-or-flight” system, while the PNS is denoted as the “rest-and-digest” system.

The EDA measures the conductivity of the skin which is modulated by the dermal sweat gland activity. Upon a relevant stimulus, the SNS becomes activated and directs the sweat glands to open and release. The increment of sweat, which contains a high quantity of electrolytes, increases the conductance at the skin surface^19^. Since eccrine sweat glands are innervated by the SNS but not by the PNS. The analysis of the skin conductance decomposes the ANS complexity by offering an unbiased view of the SNS activity (emotional and physiological functions)^20^. This has been validated in the literature with the electrodermal level increasing linearly with arousal level^21,22^. On the comprehensive meta-analysis performed by Kreibig^23^, EDA changes were reported for a set of discrete emotions. The author reports a decrease in EDA observed for non-crying sadness, acute sadness, contentment and relief. Amusement, happiness and joy were characterised by an increase in EDA and faster breathing.

On the other hand, the PPG measures the peripheral tissue’s blood volume through the absorbance of the light. When light is directed into the skin, it will be absorbed by blood, tissue and others. The level of absorption will be modulated by the blood volume and its haemoglobin constituents which vary with the cardiac cycle. During the systole, the blood volume will be higher with higher haemoglobin levels resulting in a higher absorption of light. The opposite is observed during the diastole, where the low arterial blood volume results in the PPG minimum value. These blood volume changes characterise the PPG signal (after inversion) through which metrics such as HR can be extracted. The HR is controlled by both the SNS and PNS. While the SNS is responsible for the increase in HR through vasoconstriction, the PNS is responsible for slowing it down. The combination of both modulates the HR and allows for the fine-tuned modulation and variability of the cardiac system in response to varying physiological and environmental conditions, emotional states, and stress levels.

In its systematic review, Kreibig^23^ observed that the HR increased in negative (anger, anxiety, contamination-related disgust, embarrassment, fear, crying-sadness), positive (anticipatory pleasure, happiness, joy) emotions and surprise. On the other hand, HR decreased for mutilation-related disgust, imminent-thread fear, non-crying and acute sadness, affection, contentment, visual anticipatory pleasure and suspense. The changes in the HR (HRV) have also been correlated to emotional responses. The HRV was reported to increase for amusement and joy, while it decreased for happiness. An increase in HRV was observed in contamination-related disgust and the positive emotions of amusement and joy.

The literature substantiates the physiological foundation of both EDA and PPG for emotion recognition. Particularly in daily living scenarios, potentially advancing the field of affective computing and emotion-aware technology applications.

## Methods

In light of the promising potential of EDA and PPG data for emotion recognition in an unobtrusive approach. We prepared the G-REx dataset to bridge the gap in the field between the real-world and controlled experiments, by opting for collecting data in long-duration content in a cinema, retrospectively annotated in small segments by their emotional relevance. Our approach, by not disturbing the volunteers while collecting data and using a quick and easy annotation method, facilitates the collection of large amounts of naturalistic data, leading the way for the understanding of emotional responses in real-world contexts as well as the analysis of group dynamics.

### Dataset design

**Fig. 1:**
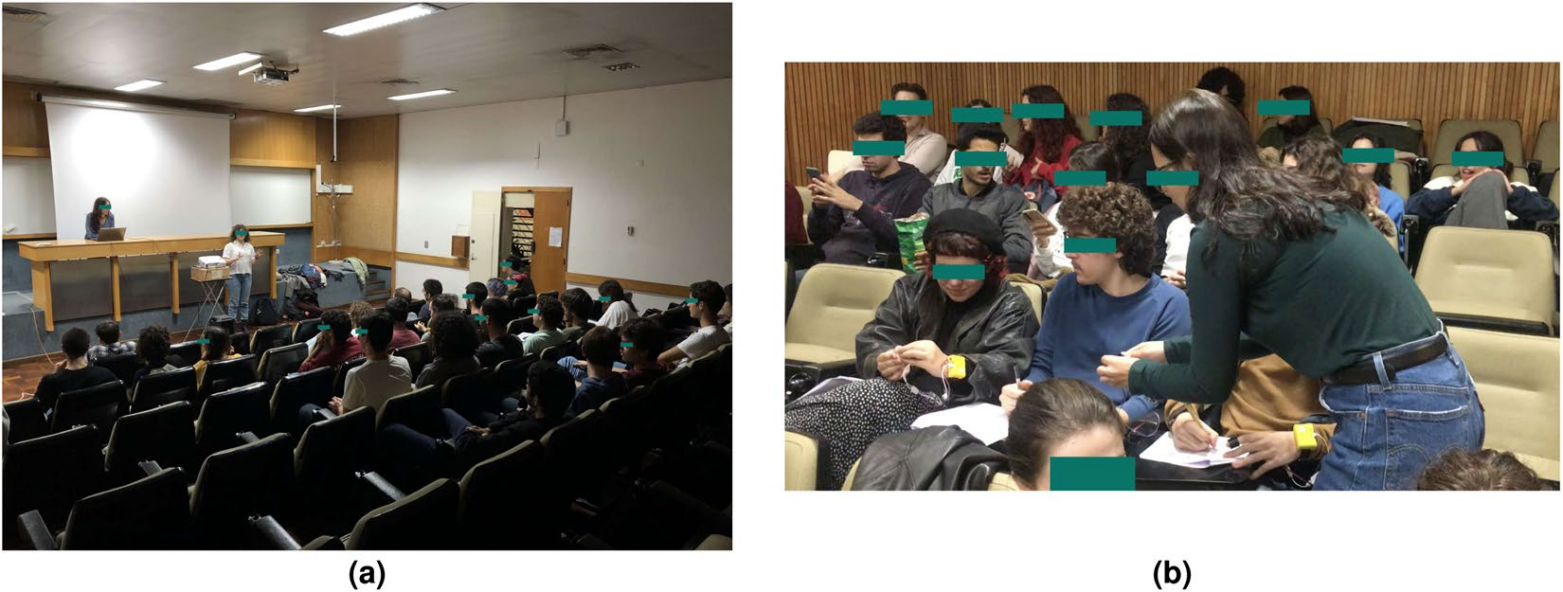
Photos taken during the movie sessions.

The G-REx dataset was designed for large data collection in naturalistic group scenarios. As a proof-of-work, we collected data at a University campus room replicating a movie theatre (Fig. 1). Instead of relying on selected movie clips as observed in the literature^5,7,11^, we rely on longer-duration content such as a cinema movie.

The dataset was collected with the following objectives:

1. Expand the annotated public datasets for physiological-based affective computing collected in naturalistic scenarios.
2. Analyse physiological-based group dynamics in a long-duration setting.
3. Propose an experimental setup that can be easily replicated across any naturalistic setting with little effort to the user based on unobtrusive physiological data collection and retrospective emotion annotation.

### Data annotation

Datasets on emotion recognition usually contain emotion-annotated segments. In the CASE dataset^6^ real-time continuous annotation of arousal and valence is performed while the participants watch videos of a few seconds (below 200 seconds). This is the literature common practice for short clips^5,6,11,24^. However, to perform the annotation in naturalistic scenarios or as in the case of a long film, the annotation becomes tiring and distracts the user from the elicitation process; Not being compatible with a naturalistic emotion elicitation.

An alternative format is performed in K-EmoCon^4^, where an approximate 10-minute debate is annotated based on three perspectives: self-reporting, by the partner, and by external observers. The annotation was performed retrospectively every 5 seconds while viewing the debate footage in terms of arousal and valence and 18 discrete emotions. The annotation through external observers can have drawbacks in a naturalistic scenario such as additional annotators becoming expensive in large-scale scenarios. Additionally, since the annotation is resorting to the observation of bodily expressions these can not truly describe the emotion felt by the subject or are subjective to the external annotation interpretation bias.

With this in mind, we follow a similar strategy to K-EmoCon^4^ (i.e. retrospective annotation), designed to better fit naturalistic settings. In G-REx, the annotation is performed by the subjects themselves and is as quick and less intrusive as possible. Instead of annotating the entire two-hour movie, which would be too time-consuming and tiresome for the volunteer, we ask for the subject to annotate only selected segments of a few seconds (20 seconds) where events (onset events with high amplitude) were detected on the subjects EDA data (segment of [onset - 5 seconds, onset + 15 seconds]). We selected a time interval of 20 seconds, following what was performed in AMIGOS^25^, and the 5 seconds previous to the onset was taken from the literature reporting the latency period of emotional stimuli between 1 to 5 seconds^26^.

We first filter and smoothen the EDA signal (Butter low-pass 4^*th*^ order and 5 cut-off frequency, 0.75 * sampling rate *boxzen* kernel smoother), then extract the EDR component and apply a smoother (20 seconds *boxzen* kernel) to the EDR so minor variations in the data would not be detected. Then, we apply the *emotiphai_eda* method from BioSPPy (https://github.com/scientisst/BioSPPy/blob/main/biosppy/signals/eda.py) and merge events with onsets separated for less than 32 seconds, so moments separated in time are selected for annotation.

The literature^27^ has shown that events with higher amplitude are easier to recall for the subject. Thus, the segments are sorted by the amplitude so the highest emotional events as given by the EDA signal are annotated first. This leads to the annotation of a lower number of segments (lower complexity) ordered by their emotional importance (more time-efficient) albeit more meaningful (hence potentially more informative).

**Fig. 2:**
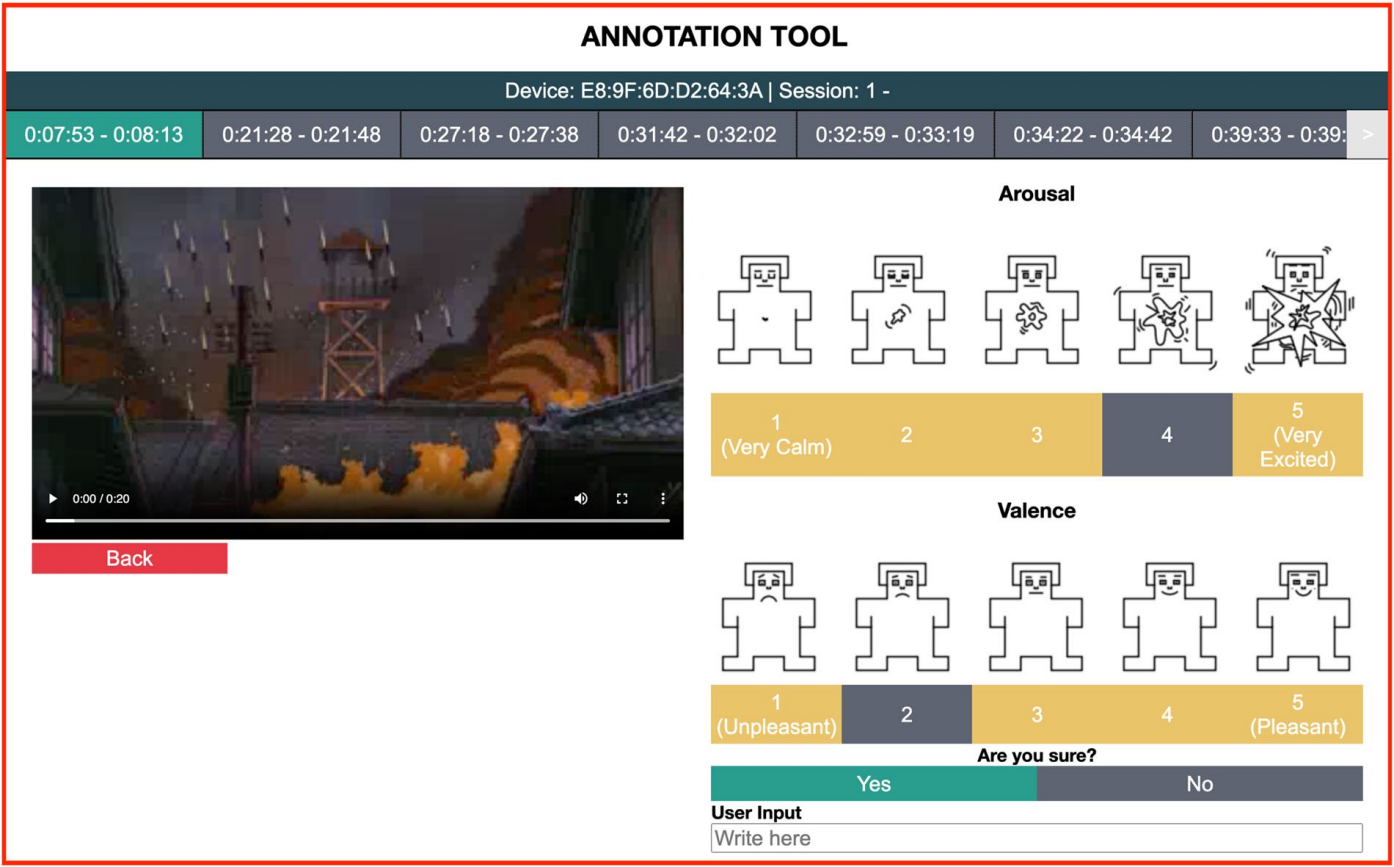
Illustration of the annotation platform.

To perform the self-report the annotation platform relies on the SAM assessment technique, the gold standard technique in the state of the art for emotional report^28^. Lastly, the literature^29,30^ reports that although past emotional events can not be re-experienced, they can be reconstructed by anchoring on relevant thoughts or events. With this in mind, during the annotation, the re-visualization of the video segments is used to provide context as an anchoring tool to help the reconstruction of the emotional events experienced by the subject. An illustration of the annotation platform used in this study is shown in Fig. 2.

### Participants recruitment

The data collection was performed between October 2022 and June 2023, making a total of 31 sessions. The volunteers were recruited from participants in the Diferencial (https://diferencial.tecnico.ulisboa.pt/) cinema sessions. Diferencial is a student club from the Instituto Superior Técnico of the University of Lisbon. The cinema sessions were advertised in Diferencial’s social media platforms, namely Twitter and Instagram being free and open for anyone to participate, regardless if they are students at the university. In the post description, there was a notification that physiological data collection was being performed on volunteers. At the cinema sessions, a lab researcher of the team approached the audience, described the experiment and asked if they would like to participate. At the end, a chocolate bar was given as a reward for the participation.

### Ethics statement

The study was submitted to and approved by the Instituto Superior Técnico (IST) - University of Lisbon (UL) Ethics Committee (Ref. n. ° 11/2021 (CE-IST) Date: 20/04/2021). The participants were given a consent form upon arriving at the cinema room before the data collection. The informed consent form contained information regarding the context, goal, procedure, data registration, data privacy and risks of participation in the experiment. In this form, the participants manually filled in their participation agreement, age, gender, if they participated in the study with any friends and their familiarity with the movie. Additionally, the participants were asked to fill out a physiological data purpose form with an agreement for the usage, visualization and analysis of the data, sharing the data in academic publications, at conferences, in media, and with external partners. Participants were notified that their participation is voluntary, must be done in an informed and free manner, and that their data can be destroyed and their participation withdrawn by request at any time without consequences.

### Data collection setup

**Fig. 3:**
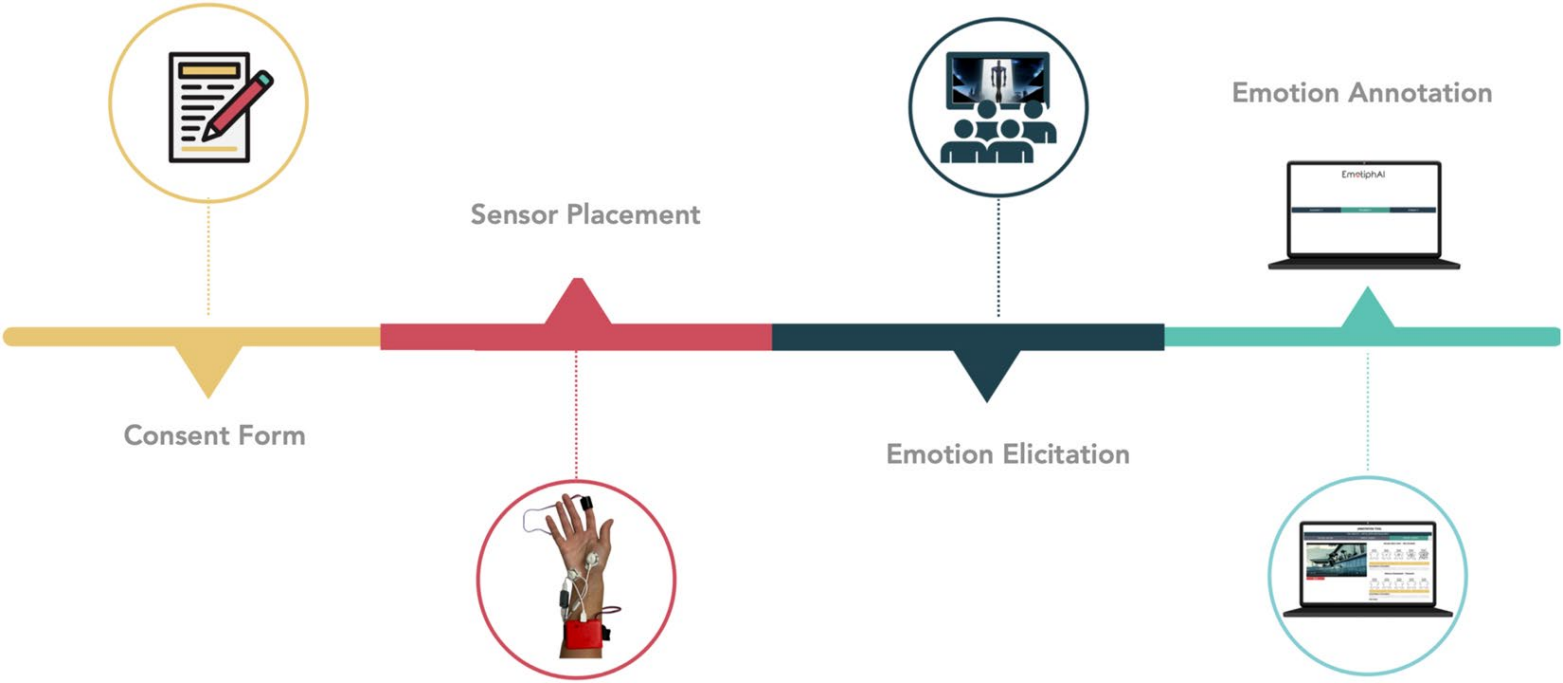
Timeline with the experimental protocol steps.

The data collection took place during the Diferencial cinema sessions, on an amphitheater at the Instituto Superior Técnico, University of Lisbon. The cinema sessions were performed once per week starting at 8 to 8.30 PM during the school academic year. The EmotiphAI^31^ platform was set up in a corner of the room, with the router at the center. During the movie visualization, the participants were given the informed consent form and the EmotiphAI bracelet was placed on their non-dominant hand with the respective sensors. The EDA sensor electrodes were placed on the thenar and hypothenar areas, while the PPG was placed surrounding the index finger distal phalange (red circle in Fig. 3). The hardware and software used for the experiment is detailed below:

**Hardware:**

- EmotiphAI collector (Microsoft Surface Pro, 7 1.10 GHz x 8 CPU): was used to host the EmotiphAI platform for both physiological data collection^31^ and emotion self-reporting.
- EmotiphAI wearable (Fig. 3): contained two physiological sensors that were connected to the subject’s skin using Pre-gelled Ag/AgCl electrodes. The details of each sensor are described in Table 1. The EDA sensor consisted of the BITalino EDA sensor (https://pluxbiosignals.com/products/electrodermal-activity-eda-sensor), and the PulseSensor PPG from (https://pulsesensor.com). The EmotiphAI Wearable was based on a modified version of the ScientISST CORE, a device designed for monitoring and analyzing physiological data (https://scientisst.com).

**Table 1:** Overview of EmotiphAI wearable characteristics.

SR: 100	Res: 12 bits	Comm: WiFi (TCP)	Range: 100 meters	Size: 22.51 × 40.90 × 53.50 (mm)	Weight: 64 g	Battery: Li-On; 7.4 V 800 mA
Sensor	Range	Bandwidth	Input Voltage Range			
EDA	0–25 *μ*S	0–2.8 Hz	1.8–5.5 V			
PPG	0.3 – Vdd		3–5.5 V			

SR: Sampling rate; Res: Resolution; Comm: Communication.
- Pre-gelled Ag/AgCl electrodes: were used to improve the skin conductivity and electrode’s adherence to the skin.
- TL-WR940N router: provided the local network for WiFI communication with no password between the EmotiphAI bracelet and the EmotiphAI collector.

**Software:**

- EmotiphAI collector: The physiological data collection was controlled by the EmotiphAI data collection platform^31^ adapted for the scientISST device. The platform allows for data storage and real-time data visualization. The data was stored locally on the hub device and then moved to a private cloud. For further detail on the acquisition platform, we refer the reader to^31^.
- EmotiphAI annotator: The annotation platform runs on the collected data on post-processing. After the movie is over, the annotation platform iterates across the users and, for each, their most significant moments as given by high amplitude EDA events (onset to peak amplitude) are selected for the volunteers to annotate retrospectively. For further detail on the annotation platform and segment selection methodology, we refer the reader to the Data Annotation section.

### Experimental protocol

Figure 3 shows the experimental procedure for physiological and emotional data collection at each cinema session. A research team of two to three members was on the site to help follow each step of the protocol.

#### Consent form

As each participant arrived for the cinema session, they were approached by one member of the research team, given a description of the experiment, and asked if they would like to participate. If they agreed, an informed consent form was given for the volunteers to read and fill out.

#### Sensor placement

Upon agreeing to the data collection, the EmotiphAI wearable was placed on the non-dominant hand with two physiological sensors (EDA and PPG). The volunteers were then ready to start watching the movie and start the data collection.

#### Data collection

The movie and physiological data collection were manually synchronised by starting both at the same time. The volunteers watched the movie in a naturalistic scenario, being able to sit in any location in the amphitheater, surrounded by their friends or strangers.

#### Data annotation

The volunteers were approached by our team to ensure the annotation of emotional segments in terms of arousal, valence and uncertainty in the annotation. A description of the emotion annotation is displayed in Table 2.

**Table 2:** Description of self-reported emotion annotation scales.

Category	Description	Range
Arousal	Denotes general energy deactivation/activation^61^	[1, 5] ∈ ℕ
Valence	Denotes displeasure/pleasure^61^	[1, 5] ∈ ℕ
Uncertainty	Denotes the level of uncertainty/certain in the emotion annotation	Yes/No

Overall, each session lasted approximately 2 hours and 30 minutes (depending on the movie length), which included around 10 minutes for emotional self-reporting, and an additional 10 minutes for sensor placement and the completion of the consent form.

#### Follow up

The literature on emotion recognition has shown that the personality type might influence the emotional reaction (e.g. neuroticism is correlated to high negative-emotional response)^32,33^. With this in mind, on the day after the data collection, a follow-up email was sent to the volunteers. The follow-up email had the goal of thanking the participants for their contribution and sharing an optional questionnaire with the big-five factor model for personality assessment using the 50-item English version of the International Personality Item Pool (IPIP-J)^34^.

### Affective stimuli

The cinema movies were selected by the Diferencial team. Most of the movies were part of thematic cycles, each dedicated to specific themes. The collected data covered the following cycles: “Horror”, “Ghibli”, “Mind*uck”, “Musical”, “Asian culture”, and “Is Anybody out there?”. Additionally, two collaborations were performed with the IST student groups (“AmbientalIST”, “NucleAr” and “AEIST”) and one collaboration with a production company (“JumpCut”). Before two of the sessions, a short film was displayed before the movie data collection.

## Data Records

The G-REx dataset is available on *Zenodo* (https://zenodo.org/record/8136135)^35^. The data will be made available after completing the End User License Agreement (EULA) (available at https://forms.gle/RmMosk31zvvQRaUH7).

The G-REx dataset was organized to contain both the Raw and Transformed data. As well as all the code for the data transformation. To guarantee the reproducibility and transparency of the research, the code utilized for the data transformation is also included within the dataset, with the resultant plots and table content derived during the research process.

### Dataset contents

**Fig. 4:**
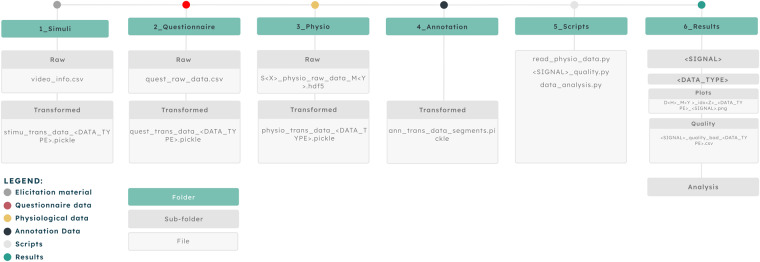
Diagram of the dataset structure.

The G-REx dataset is available on *Zenodo* (https://zenodo.org/record/8136135). For the structure of the dataset, we followed a similar approach to the one in^36^. An overview of the structure is shown in Fig. 4. The dataset contains six main folders, outlined as follows:

- 1_Stimuli:

- Raw/video_info.json/csv — Contains detailed information on the movies used in the dataset.
- Transformed/stimu_trans_data_<*DATA_TYPE*>.pickle — Contains the information of the movie details for the annotated segments and session data. <*DATA_TYPE*> ∈ {session, segments}.

- 2_Questionnaire:

- Raw/quest_raw_data.json/csv/xlsx — Contains the questionnaire data for all the participants in the dataset.
- Transformed/quest_trans_data_<*DATA_TYPE*>.pickle — Contains the user ID and device information of the emotion annotated segments and session data. <*DATA_TYPE*> ∈ {session, segments}.

- 3_Physio:

- Raw/S<*X*>_physio_raw_data_M<*Y*>.hdf5; where <*X*> is the session ID ∈ {0, 1,…, 28}, and <*Y*> is the movie ID ∈ {0, 1,…, 30} — Contains the raw HDF5 data collected by the EmotiphAI platform for each session *X* and movie *Y*.
- Transformed/physio_trans_data_<*DATA_TYPE*>.pickle – Contains the transformed raw and filtered EDA, PPG, HR and time information data for the annotated segments and session data. <DATA_TYPE> ∈ {session, segments}.

- 4_Annotation:
  - ann_trans_data_segments.pickle – Contains the arousal, valence and uncertainty values for the annotated segments.
- 5_Scripts:

- read_physio_data.py – Reads the collected raw physiological signals, emotion annotations, video data and the self-report questionnaires to store the transformed data in separate dictionaries, with matrices data for the session and annotated data segments.
- data_analysis.py – Script used to obtain the plots and tables displayed in the technical validation section.
- quality_<*SIGNAL*>.py – Obtain the data quality and lower quality signals. <*SIGNAL*> ∈ {EDA, PPG}.
- 6_Results: <*SIGNAL*>/<*DATA_TYPE*>/Plots/D<*H*>_M<*Y*>_idx<*Z*>_<*DATA_TYPE*>_<SIGNAL>.png – Plot of the raw and filtered data. The<H>variable is the device ID, <Y> is the movie name, and <Z> is the sample index. <SIGNAL> ∈ {EDA, PPG}; <DATA_TYPE> ∈ {session, segments}. <SIGNAL>/<DATA_TYPE>/Quality/<SIGNAL>_quality_bad_<DATA_TYPE>.csv – Table with the physiological signals technical validation results.<SIGNAL> ∈ {EDA, PPG}; <DATA_TYPE> ∈ {session, segments}.

**Fig. 5:**
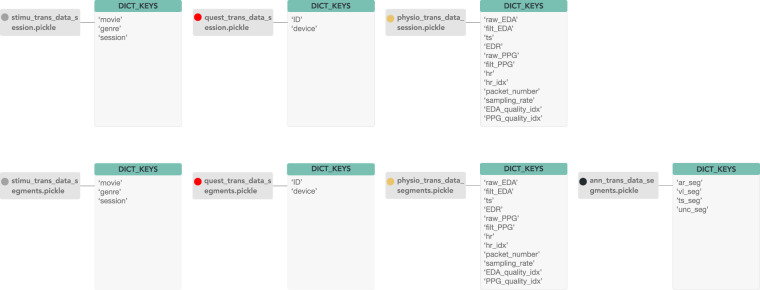
Diagram showing the structure of the pickle data in their respective keys.

Each pickle file consists of a dictionary with several keys. The detailed information regarding the components of each pickle file can be seen in Fig. 5.

### Dataset summary

The G-REx dataset consists of data collected from 31 sessions, making a total of 191 users and more than 380 hours of data collected. This includes data from the physiological signals EDA and PPG, and emotional annotations of selected movie moments, making a total of + 1400 annotated segments. In two of the sessions, two different movies were seen, while in the remainder only one movie was seen resulting in a total of 31 sessions but 29 movies.

Lastly, we also gathered the movie file for context information. A summary of the total collected data is displayed in Table 3.

**Table 3:** Summary of the dataset data.

	Total	After Processing		Segments
	Session	Segments	Session	
# of Participants	191	191	92	149
# of Movies	31	31	27	27
# Sessions	29 (≈384.5 hours)	29 (≈8.2 hours)	25 (≈175.9 hours)	25 (≈5.7 hours)
# Samples	241	1481	112	1031
Age Range		18–69		
Physiological Signals		EDA, PPG		
Emotion Annotations		Arousal, Valence		

#### Preprocessing

The raw data was collected using the EmotiphAI collector and stored on an *HDF5* file^37^ (fit to store large amounts of data in a hierarchical structure with several databases, i.e. for various devices on the same session containing both physiological and emotion annotations). To facilitate the usage of the data, we provide a compact format of the session and segments data, as well as questionnaire, stimuli and emotion annotations on separate but synchronized dictionaries stored on *pickle* files.

## Technical Validation

To characterise the collected data, we provide a quality evaluation of the dataset data. We start by looking at the physiological data, dividing it into the quality and lower-quality sets, and looking at description metrics to see how the two compare. We then observe the distribution of emotion annotations and movie genres, underscoring the dataset’s representation. Lastly, we performed statistical evaluations, comparing the distributional properties of arousal, valence, and genre groups, benchmarked against mean EDA, HR, and subjectively reported arousal and valence measures.

### Physiological signals

The EDA signal is read through the connection of gel electrodes to the skin. This makes the EDA easily subjected to artifacts such as loss of contact of the electrodes with the skin either due to a long-duration use or sweat, which disconnects the gel glue. These artifacts can be removed using a low-value threshold. A similar analysis can be performed to the PPG signal, where the sensor can lose connection to the finger or be too tight causing saturation at the maximum value. In addition, the PPG allows the extraction of the HR, which has expected range-values denoted in the literature between 40 and 200 bpm^38^. Values outside this range are noise and can be removed.

To remove noisy data and obtain a view of its description, we applied quality metrics identified in the literature^31,38–43^ for the EDA and PPG signals. Using the quality metrics, the data was divided into quality and lower-quality sets. As observed in the literature, some works apply cut-offs to remove low-quality data using a cut-off threshold^40^, while others^44^ use the metrics to obtain an overview of the data distribution of the two to observe how the quality segments and lower quality segments distributions compare to each other.

Additionally, our technical validation of the physiological signals is performed for the two types of data collected: the entire data in a session format, and 20-second annotated segments. A 4-second threshold was used following^40^ to denote the lower-quality data. For the session data, which contains data around 2 hours, the 4 seconds was meaningless, we used a conservative approach and relied on a threshold of 7% for the session data.

### Electrodermal activity

The experimental results for the data quality analysis on the sessions and emotion segments can be seen in Table 5 for the EDA data.

#### SNR

The experimental results show a SNR of around 120 dB for the EDA signal, meaning that the signal level is approximately 120 dB higher than the noise level for both the session and the annotated segments data. The SNR decreases for the lower quality data, with values around 100 dB for the sessions and even lower for the segments data (≈40 dB), both showing higher standard deviation in the lower quality data. In^45^ the authors report a SNR between ≈50 to 60 dB. While in^5^, the authors report an average SNR between 26.66 dB to 37.74 dB (reporting for all the collected signals). These values are in line (or even below) with what was obtained in our work for the quality data set. However, it is expected for these values to change according to how the SNR metric was obtained. We followed the approach by^42^, where a SNR value was obtained between 50 to 80 dB. Similar values were obtained by the authors in^44^, namely 61.6 dB for the EDA.

#### Full scale

Across all the data we observe no saturation. Saturation is commonly observed when the sensor range of values is below the reading, for example by a hard press on the sensor. The results show a correct positioning of the sensor, with data in line with the physiologically expected.

#### Zero

The zero percentage allows the detection of records where no data was collected. The percentage of zero-valued signals is low, attaining a value below 1% for the quality data in both the sessions and annotated segments. On the other hand, we observe a high percentage of zero data (%) on the samples identified as lower-quality data (above 50%). This shows that the zero-data metric is the most contributing factor for the assignment as lower quality data. The zero value read by the sensor can be due to the loss of contact between the electrodes and the skin, physiological problems such as hyperhidrosis where the sweat causes the electrodes to disconnect after a long session (such as it can happen in a 2-hour movie), or problems related with the device form-factor (for example a cable was set loose or broken). Additionally, it should be taken into consideration that due to the naturalistic setting of the data collection, some of the devices lost WiFi connection (e.g., volunteers leaving the room), leading to no data being recorded.

The literature^46^ expects a prevalence of hyperhidrosis at 4.8%, following a study in the USA population in 2016. However, this value might be biased towards a severe case, with 70% reporting severe excessive sweating. In our study, a hyperhidrosis percentage above the expected can be because our proof of work device relies on the use of gel electrodes to connect the sensor to the skin, and 2 hours of movies can be too long for the electrodes to stay in place.

#### Loss

Across the dataset we observe a data loss of 0%. Our data loss is much lower than the reported in the literature, such as in the work of^41^, where the authors report a data loss of around 50% on average for data streaming. Similar to our values, a loss of 0% is reported in^44^.

#### Max, Mean, Min

The lower quality data in both the sessions and annotated segments show a lower average value (around 0 *μ*S). Similarly, the maximum and minimum values are higher in the quality data when compared to the lower-quality data.

Through these statistics, we can see if the EDA data is in line with what is reported in the literature. Namely, Braithwaite *et al*.^47^ reports expected EDA values between 2 to 20 *μ*S, increasing for periods of high arousal. In^48^, the authors report EDA data between 10 and around 28 *μ*S (M (Mean): 15.55; STD (Standard Deviation): 1.67). These values are in line with the values obtained for the quality data, taking that deviations can result from the type of electrodes and their body location.

### Photoplethysmography

A similar analysis was performed for the PPG data. The quality metrics can be seen in Table 4, and the obtained results in Table 6.

**Table 4:** Data quality metrics deployed for analysing the EDA and PPG signal quality.

Signal	Metric	Description
Cut-Off		
EDA & PPG	Full scale	Amplitude at the bit resolution of 12 bits (2^12^—1) for more than 4 seconds and 7% in the session data^39^. Applied to the filtered data.
EDA & PPG	Zero	Amplitude below 0.05 *μ*S for EDA and 0.01 a.u. for the PPG for more than 4 seconds in the segment data and 7% in the session data. Applied to the raw data.
EDA & PPG	Loss	Counting packet number and time discontinuities^31^. Data was considered as lower quality if it had a data loss above 7%.
PPG	Abnormal Heart Rate (HR)	HR below 40 bpm or above 200 bpm detected for more than 4 seconds in the segments or 7% in the session data was considered as low-quality data^38^.
Data Distribution		
EDA & PPG	Signal-to-noise Ratio (SNR)	For the EDA, SNR was obtained by the logarithmic ratio between the cleaned signal (low-pass 4^*rd*^ order Butterworth filter of 5 Hz and 0.75 seconds-window smoother moving average)^60^ and noise (3^*rd*^ order Butterworth band-pass filter of 2–10 Hz)^42^. For the PPG, the noise was obtained by a 4^*th*^ order highpass Butterworth filter with 15 cut-off frequency^62^ and the cleaned signal by a 4^*th*^ order band-pass Butterworth filter with 1–8 cut-off frequency^60^.
EDA & PPG	Max, Mean, Min	Statistical features (maximum, mean and minimum) extracted from the filtered data^39^.
PPG	Spectral Entropy	.Entropy of power spectrum between 0.1 to 3 Hz, ranging between 0 for a periodic signal and 1 for a constant spectrum^40,41,43^

**Table 5:** Data quality metrics analysed for the EDA signal.

Data	Size	SNR (db)	Full scale (%)	Zero (%)	Loss (%)	Max (*μ*S)	Mean (*μ*S)	Min (*μ*S)
Quality Segments	1265	122.71 ± 23.26	0.0 ± 0.0	0.3 ± 1.84	0.0 ± 0.0	7.85 ± 5.22	6.42 ± 4.25	5.48 ± 3.89
Lower Quality Segments	216	37.79 ± 35.3	0.0 ± 0.0	83.46 ± 22.4	0.0 ± 0.0	2.94 ± 5.3	0.7 ± 1.71	0.0 ± 0.01
Quality Session	136	127.12 ± 15.09	0.0 ± 0.0	0.75 ± 1.7	0.0 ± 0.0	15.54 ± 6.59	7.06 ± 4.3	2.03 ± 3.41
Lower Quality Session	105	100.56 ± 39.71	0.0 ± 0.0	52.06 ± 32.3	0.0 ± 0.0	9.97 ± 7.51	1.75 ± 1.96	0.0 ± 0.0

**Table 6:** Data quality metrics analysed for the PPG signal.

Data	Size	SNR (dB)	Full scale (%)	Zero (%)	Loss (%)	Abnormal HR (%)	Spectral Entropy	Max (a.u.)	Mean (a.u)	Min (a.u.)
Quality Segments	1213	76.07 ± 14.24	0.01 ± 0.17	1.62 ± 3.16	0.0 ± 0.0	0.0 ± 0.0	0.68 ± 0.09	1903.95 ± 1137.23	24.09 ± 55.37	−1244.47 ± 745.14
Lower Quality Segments	268	3.6 ± 14.39	0.03 ± 0.32	95.57 ± 17.61	0.0 ± 0.0	93.66 ± 24.37	0.05 ± 0.18	196.01 ± 809.31	1.65 ± 15.63	−150.52 ± 607.29
Quality Session	199	72.47 ± 9.92	0.01 ± 0.08	1.21 ± 1.55	0.0 ± 0.0	0.0 ± 0.0	0.72 ± 0.07	3518.95 ± 1122.89	14.93 ± 27.25	−2539.92 ± 774.41
Lower Quality Session	42	7.92 ± 20.47	0.01 ± 0.05	89.37 ± 28.96	0.0 ± 0.0	85.71 ± 34.99	0.10 ± 0.25	605.43 ± 1543.48	1.72 ± 6.01	−432.49 ± 1104.39

#### SNR

The difference in the SNR in the noisier and quality data sets are predominant, with the quality data showing a SNR above 70 dB and the lower quality data SNR below 10 dB.

#### Full scale

Once again no saturation is observed. Similarly to^39^, we obtained a very low full-scale percentage.

#### Zero

We observe a higher number of zeros in the lower-quality data in both the sessions and the annotated segments, while the quality data has a lower percentage of zeros (below 2%). In some of the sessions, some of the devices were turned on but were not being used by any volunteer, thus recording a value of 0 throughout the entire session.

#### Abnormal HR

Abnormal values for the HR (below 40 and above 200^38^) are not observed in the quality data. While for the lower-quality data, abnormal values are observed for almost the entire set (around 90%).

#### Spectral entropy

We observed higher entropy for the quality data. The authors in^40^, denote a lower entropy is expected for the quality data, denoting a pointier spectrum in its amplitude waveform. While a flat spectrum (i.e. uniform distribution) is characterised by higher entropy and noisier data. This was not observed in our data. The higher entropy observed can be a result of the increased complexity in the quality data which shows higher standard deviation and diverse morphology compared to a flat line (lower quality data signal). Our results are in line with^41^, which reports a threshold of around > 0.8 to denote a lower-quality signal.

#### Max, Min, Mean

The data shows lower amplitude values for the lower data quality set, attaining a minimum value of zero.

Overall, we observe that, for the EDA sensor, the annotated segments show a smaller proportion of lower-quality segments compared to the session data. A large amount of data was removed from the session set due to the presence of zero-value data for more than 7% of the session. The large amount of data being selected as zero results from the small threshold used as a criterion to identify the data was low quality (7%) in around 2 hours of data collection. During a 2-hour session, the individuals are likely to get relaxed/bored and the EDA drops to zero episodically.

While for the segment data, the threshold was set as 4 seconds in 20 seconds of data. These values were selected following the literature^40^. The authors in^40,41^ report around 64% to 75% of quality EDA data. We obtained around 85% for the segments and around 55% for the session data. While for the session data, large amounts of EDA data were discarded due to the zero-metric, the same was not observed for the PPG data. Leading to a lower number of sessions and annotated segments being identified as lower quality. For the PPG data, the authors in^41^ report around 50% of quality data using an Empatica device. We obtained around 80% for the segments and session PPG data.

### Data characterisation

After the pre-processing step of the noisy sample removal, we obtained a total of 1031 annotated segments and 112 sample sessions.

**Fig. 6:**
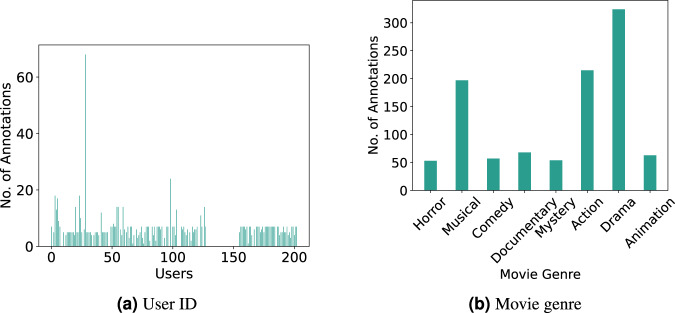
Histogram with the number of annotations per user and samples collected per movie genre.

A histogram with the total number of annotated samples can be seen in Fig. 6a. The figure shows that overall the users annotated around 5 to 10 samples per session. The number was tailored to 7 following the volunteer’s feedback on their preferences. With a few exceptions of users who participated in more than one session. For example, one subject who was part of the cinema club participated in most of the sessions, which is seen by the large peak in the histogram near ID 25. The IDs in the blank correspond to users who were removed on the data pre-processing step or did not annotate any segments.

Each movie was assigned to its predominant genre following the *IMDB* characterization (https://imdb.com). In Fig. 6b we show the assigned movie genre of each annotated sample. As can be seen, due to the movie cycles, we collected data from eight main movie genres.

**Fig. 7:**
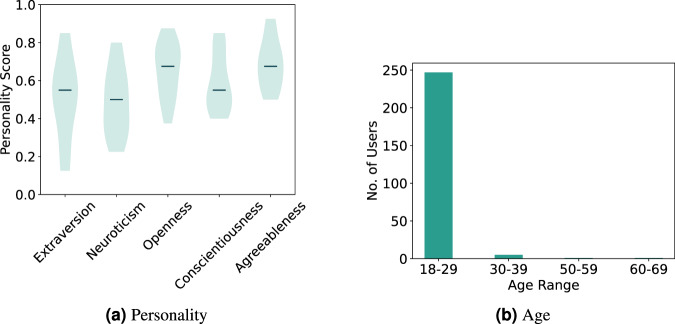
Distribution of personalities and age range.

Figure 7a displays the volunteer’s personality scores across the big five dimensions. The extraversion dimension shows a broader range of values covering most of the scale, followed by neuroticism. These dimensions have been correlated to the expression of emotions, namely to the frequency and intensity of positive and negative emotions^49^. The remaining dimensions are skewed to the upper range of the scale.

Lastly, Fig. 7b shows the age range of the volunteers. As can be seen, we acquired data across all age distributions, with 18 to 29 years old being predominant. This range is expected since the data collection took place on a university campus.

### Emotion annotation

**Fig. 8:**
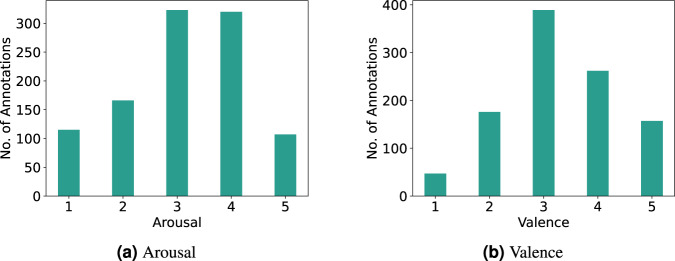
Histogram of the self-reported annotations.

After the movie, the EmotiphAI^31^ platform was used to annotate selected movie scenes using the SAM^50^ manikins on the arousal and valence dimensions. The results of the annotations can be seen in Fig. 8, where both dimensions cover the entire annotation space. Statistical characterization of the annotations distribution is shown in Table 7. As can be seen, both dimensions are centered around the value of 3, with a standard deviation of around 1. Both dimensions show negative (left-modal) skewness and kurtosis. The valence shows a near-zero skewness corresponding to a symmetrical distribution, while arousal is slightly negatively skewed. Regarding the kurtosis score, both dimensions show an elevated negative kurtosis score. A high negative value describes a flatter distribution compared to a normal distribution, denoting a more homogenous distribution of the data across the annotated scale with the probability of values near the mean lower than in a normal distribution. Moreover, the distribution has lighter tails, suggesting fewer extreme values. The authors in^11^, also analyze the annotations of kurtosis and skewness in the technical validation, where an overall negative skewness and a positive kurtosis distribution are obtained for valence and arousal. However, it should be taken into consideration that these metrics are heavily impacted by the number of samples and the elicitation content.

**Table 7:** Statistics on the annotations distribution.

	Mean	STD	Skewness	Kurtosis
Arousal	3.13	1.15	−0.29	−0.68
Valence	3.30	1.06	−0.08	−0.57

### Statistical analysis

A statistical test for a normal distribution of the different groups was computed using the Shapiro test (https://docs.scipy.org/doc/scipy/reference/generated/scipy.stats.shapiro.html), to test the null hypothesis that the data was drawn from a normal distribution. When any of the obtained p-values are below the thresholds for significance level (i.e. 0.05) we rejected the null hypothesis and concluded that the data is not normally distributed. Taken that the data is not normally distributed, we computed the Kruskal-Wallis H-test (https://hdocs.scipy.org/doc/scipy/reference/generated/scipy.stats.kruskal.html#scipy.stats.kruskal) to assess the null hypothesis that the population median of all of the groups is equal. On the other hand, when the p-value is above 0.05, not showing enough evidence to reject the null hypothesis that the data was drawn from a normal distribution, we performed the ANOVA test (https://docs.scipy.org/doc/scipy/reference/generated/scipy.stats.f_oneway.html). All the tests were computed using the annotated segments data.

The experimental results are shown in Table 8. Analyzing the Shapiro test, across most groups, a p-value was obtained below the 0.05 threshold, denoting that the groups do not follow a normal distribution. Our results are in line with the normality test performed in^11^, where a Kolmogorov-Smirnov normality test p-value below the threshold was obtained across participants.

**Table 8:** Statistical test p-values determining whether there are statistically significant differences among the different groups.

Group	Measurement	Shapiro	Kruskal-Wallis ANOVA
Arousal	Mean EDA	(0.00, 0.00, 0.00, 0.00, 0.87)	0.17
	Mean HR	(0.00, 0.00, 0.00, 0.00, 0.00)	0.22
Valence	Mean EDA	(0.00, 0.00, 0.00, 0.00, 0.00)	0.15
	Mean HR	(0.01, 0.00, 0.00, 0.00, 0.00)	0.55
Genre	Mean EDA	(0.23, 0.51, 0.01, 0.80, 0.00, 0.00, 0.02, 0.0)	**0.00**
	Mean HR	(0.22, 0.19, 0.08, 0.00, 0.00, 0.00, 0.07, 0.00)	**0.00**
	Arousal	(0.00, 0.00, 0.00, 0.00, 0.00, 0.00, 0.00, 0.00)	**0.00**
	Valence	(0.00, 0.00, 0.00, 0.00, 0.00, 0.00, 0.00, 0.00)	**0.00**

The tested groups are arousal and valence dimensions ∈ {1, 2, 3, 4, 5}; Movie genre: Drama, Animation, Musical, Comedy, Horror, Mystery, Action, Documentary. The most significant results are shown in bold (p-value < 0.05). A Kruskal-Wallis test is performed when the normality test (Shapiro) obtains at least for one group a p-value < 0.05, and the ANOVA if all the p-values > 0.05. Tests were computed using the annotated segment data.

For the group differences (Kruskal-Wallis/ANOVA), it can be seen that overall, we fail to reject the null hypothesis of equal medians across groups (p-value > 0.05). We observe that the normalized mean EDA and HR have very similar medians across the different groups, i.e. arousal and valence scores 1 to 5. These results are expected since emotion classification is a complex task and a more diverse set of features (rather than the mean of the signals), combined with artificial intelligence algorithms are required to separate the different classes and perform emotion recognition.

An exception is the normalized mean HR and EDA, and the arousal and valence self-reports for the different movie genres where a p-value below the threshold was obtained. Denoting that at least one group’s population median is different from the others.

**Fig. 9:**
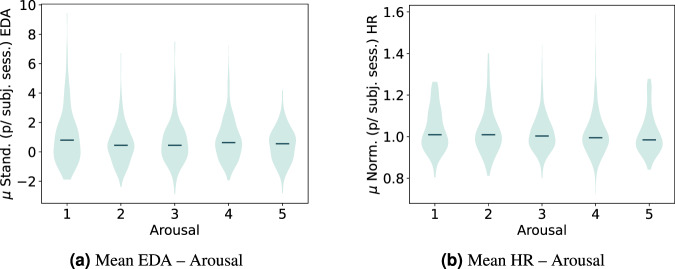
Violin plot distribution for the mean standardized EDA (subtracting the session mean and dividing by the session standard deviation per subject) and normalized HR (dividing by the session mean per subject) across the arousal self-report scores. The group medians and extrema are shown. Test computed using the annotated segments data.

**Fig. 10:**
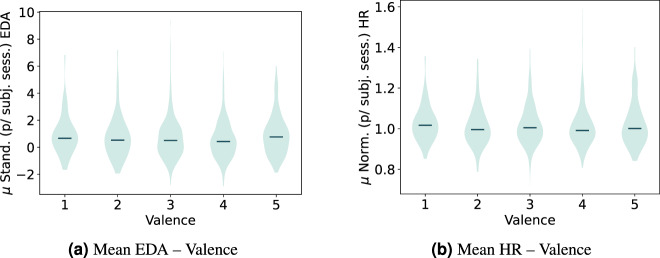
Violin plot distribution for the standardized mean EDA and normalized HR across the valence self-reported scores.

**Fig. 11:**
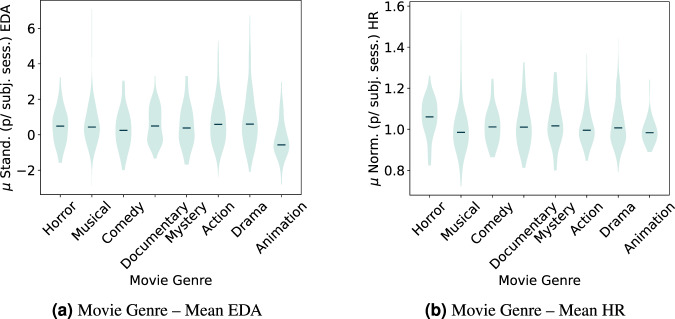
Violin plot distribution for standardized mean EDA data per subject and normalized mean HR per subject across the movie genres.

**Fig. 12:**
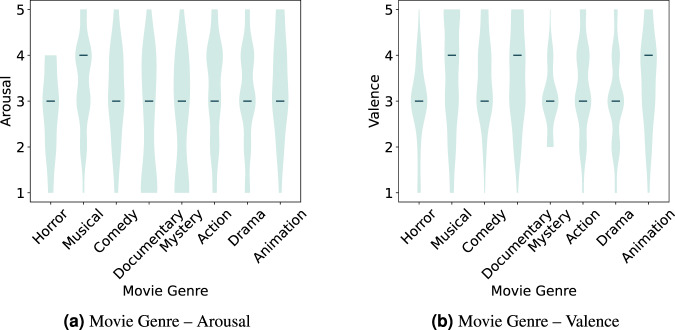
Violin plot distribution for the arousal and valence self-reports across the movie genres. Test computed using the annotated segments data.

To better understand the statistical results from Table 8, we illustrate the data distributions of the groups across the studied measurements in Figs. 9–12.

## Usage Notes

Due to access controls, we cannot directly share the videos as a part of the dataset. With this in mind, we made available a detailed description of the videos used in the “video_info.csv” file, so they can be identified by the users.

### Potential applications

The G-REx dataset provides physiological data (namely EDA and PPG) recorded during a naturalistic emotion elicitation setup. Thus, the main usage of the dataset is to explore emotion recognition analysis through physiological data closer to naturalistic data. The naturalistic scenario allowed us to acquire large amounts of data which can enable the implementation of deep learning approaches that depend on a large number of samples for a good performance.

Taking that the data was collected during movie visualizations. For the entertainment industry, such data can be used to gauge the emotional impact of movies, video games, or other media content. With the collected movie genre information, our data can be used to understand the viewers’ emotional responses to different genres and better understand viewer preferences for the development of personalized content recommendation systems. This information can assist producers and directors in refining their content to better evoke the desired emotional responses from the audience, leading to more engaging and immersive experiences.

The group context in which the data was collected has been shown to improve the emotion recognition classification in comparison to an individual-based classification^51^. Thus, G-REx group data can be used as an environment context to improve emotion classification. In a group setting, physiology synchrony has been shown that it can identify relevant events in time^52^, or important structural moments in a live concert^53^, just to name a few examples. With this in mind, our data can be used to analyze physiological synchronization, use it as context for emotion classification, identify major plot moments throughout the movie, or study inter-subject causality through physiological data. The creation of physiological synchrony indexes can be transferred to other applications such as romantic attraction^54^, learning success^55^, psychotherapy^56^, moments of connection^57^, among others.

In another field, the collected demographic information, such as personality and age, can be used as fairness constraints in algorithms to account for bias and manipulative content^58,59^.

Lastly, in addition to the intended usage of the dataset, the collected physiological data can be used to analyze physiology-related quality indices or outlier detection algorithms.

The diverse applications of physiological data such as EDA and PPG in affective computing hold significant promise for advancing fields like psychology, neuroscience, and human-computer interaction, among others.

### Limitations

The different parts of the data collection protocol and their required software/hardware are susceptible to specific constraining factors, which we detail below. The dataset limitations are related to collecting a dataset in a naturalistic scenario.

#### Data collection setup

Taking into consideration that the data collection was performed in a naturalistic setting, the volunteers were free to cover themselves and the devices with their clothes such as jackets and even leave their seats. Moreover, the movie was played by the cinema club on a separate projector. So it was necessary to start the movie and the data collection with a manual clue that directed the simultaneous start. This may present issues in the precision of timing that should be noted. However, these are not a major concern given the timescales of the physiological responses measured. Nonetheless, future work should focus on using an automatic method for synchronization.

#### Emotion annotation

Emotion annotation was performed retrospectively based on the subject emotional events read by their physiological data. Taking into consideration the naturalistic setting of the data collection, some of the detected events may not be related to emotional events but to random movements. Additionally, when performing the emotion annotation the volunteers were given a video preview of the selected moments for annotation. However, as the volunteers were freely viewing the video, their emotion elicitation could have been not from the movie but from conversations with their peers. To gather input on these issues, in the emotion annotation platform, we provide an open text box where the participants can introduce long textual external comments for each video segment, and we record the information on which participants participated in the experiment and sat side by side.

#### Participants health and room conditions

Our work aims to display a data collection methodology that can be replicated at a large scale in the real world. As such, in our proof-of-work, the volunteers were in a cinema session and we were required to collect as little data as possible so the interruption to the normal cinema session would be minimal and a large number of volunteers would be open to participate regularly in the data collection. Consequently, no records were obtained about any psychiatric or neurological conditions of the participants, nor was there any information gathered on whether participants had consumed any pharmacological medication during or prior to the study. Similarly, no data was acquired on the humidity, temperature of the room, or food intake during or prior to the movie. Such factors can potentially influence physiological responses and thereby emotional status and future works should contemplate incorporating this information if the protocol setup allows reducing this limitation while maintaining the efficiency of the data collection process.

## Data Availability

The raw data in “xlsx” or “HDF5” format was transformed into dictionaries containing the relevant data in matrices stored in a “pickle” format. For the physiological data pre-processing, we rely on the “biosppy” library^60^, which contains modules for filtering the EDA and PPG signals, peak extraction and EDA decomposition into the EDR and EDL components. For the statistical tests analysis, we used the “SciPy” library (https://github.com/scipy/scipy). For further information regarding the raw or transformed data, code incompatibilities, or others, we welcome the reader to contact our corresponding author. The processing was done in Python 3.7.4, and the required code is available in the “5_Scripts” folder on *Zenodo* (https://zenodo.org/record/8136135) so it can be easily replicated.
